# Eczema Capitis

**Published:** 1874-11

**Authors:** 


Eczema Capitis.—Cure it if possible, notwithstanding the somewhat prevalent idea to the contrary. The method of procedure recommended is as follows:

First, apply a poultice every night, until all the scabs are removed. The ulcerations, which are sometimes present after the scabs have been removed, are best cured by the application of a wash made of nitrate of silver, grs. v. to the ℥ i. of water. The following is then used with good success:

| | |
|---|---|
| ℞.—Aquæ Cologne, . . . . . . | ℥ iv. |
| Glycerine, . . . . . . . | ℥ ij. |
| Carbolic acid crystals, . . . . . | ʒ i. |
| Borax, . . . . . . . . | ʒ i. |

M. Continue the application of this remedy for some time for the purpose of curing the eczema.—*Druggists' Circular.*

